# The Patient Experience of Integrated Care Scale: A Validation Study among Patients with Chronic Conditions Seen in Primary Care

**DOI:** 10.5334/ijic.4163

**Published:** 2018-10-12

**Authors:** Heithem Joober, Maud-Christine Chouinard, Jenny King, Mireille Lambert, Émilie Hudon, Catherine Hudon

**Affiliations:** 1 Université de Sherbrooke, CA; 2 Université du Québec à Chicoutimi, CA; 3 Picker Institute Europe, GB; 4 Centre intégré universitaire de santé et services sociaux du Saguenay-Lac-Saint-Jean, CA

**Keywords:** Integrated Care, Chronic Disease, Primary Care, Questionnaire, Validation Studies

## Abstract

**Introduction:**

Valid and comprehensive instruments to measure integrated care are required to capture patient experience and improve quality of patient care. This study aimed to validate the Patient Experience of Integrated Care Scale (PEICS), among patients with chronic conditions seen in primary care.

**Methods:**

One hundred and fifty-nine (159) French-speaking adults with at least one chronic condition were recruited in two family medicine clinics in Quebec (Canada) and completed the 17-item PEICS (T1). Fifty (50) participants completed it a second time 2 weeks later (T2). The internal consistency of the scale was assessed using Cronbach’s alpha, the test-retest reliability with the intraclass correlation coefficient (ICC), and concurrent validity using three dimensions of the Continuity of Care from Multiple Clinicians (CC-MC), with Spearman’s rank correlation coefficients.

**Results:**

Cronbach’s alpha for the questionnaire was 0.88 (95% CI: 0.85 to 0.91). The intraclass correlation coefficient was 0.81 (95% CI: 0.64 to 0.90) and Spearman’s rank correlation coefficient with the three dimensions of the CC-MC varied from 0.44 to 0.54.

**Conclusions and discussion:**

The PEICS showed good psychometric properties. This scale could be used in a population with chronic conditions followed in primary care to measure patient experience of integrated care.

## Introduction

A large proportion of the population from industrialised countries suffers from chronic conditions [1]. These conditions usually persist over long periods of time, requiring long-term care from multiple service providers who handle patient needs ranging from physical and medical care to social, economic and psychologic support [2,3,4,5]. Integrated care among all healthcare and social care providers is essential to provide optimal care for these patients [6].

Integrated care is the management and delivery of coordinated and patient-centered healthcare services [7,8,9]. It includes actions aiming to improve coordination, cooperation, continuity, and collaboration across different sectors of the healthcare system to prevent duplication and fragmentation of care while placing patients at the center of their care [10,11]. Integrated care reduces hospital admissions, enhances adherence to treatment and compliance to medication, and improves quality of care, health-related quality of life, functional health, as well as patient satisfaction [6,12].

The National Collaboration for Integrated Care and Support in the United Kingdom developed a model of integrated care based on patient experience [8]. This model includes six dimensions: (1) consideration of patient and family needs; (2) communication with the patient and between practitioners; (3) access to information; (4) involvement in decision-making; (5) care planning; and (6) transitions between various health professionals and practitioners.

Valid measures of integrated care may provide useful follow-up indicators of efforts to improve quality of patient care [13]. While a few instruments have been designed to assess patient perception of integrated care [13,14,15], they do not cover all dimensions of the National Collaboration for Integrated Care and Support model. To fill this gap, Picker Institute Europe and the University of Oxford were commissioned by the Department of Health and Social Care in the UK to develop a set of items to measure integrated care. These items could be used to help understand success in achieving the goals of the National Collaboration for Integrated Care and Support model, [16] but these items have not been validated yet.

This study aimed to validate the Patient Experience of Integrated Care Scale (PEICS), based on items proposed by Picker Institute Europe and the University of Oxford, among patients with chronic conditions seen in primary care, more specifically to evaluate its internal consistency, test-retest reliability, and concurrent validity.

## Methods

### Development of the Patient Experience of Integrated Care Scale (PEICS)

To determine how services should be delivered for people with complex conditions, the National Voices in England adopted in 2013 a “narrative” for person-centred coordinated care, i.e. a new definition of integration reported in the form of statements formulated from the position of the patient [17]. Known as the “I” statements, this narrative was built on the experience of patients, healthcare providers, as well as their representative organizations and included 38 statements to illustrate what good integrated care might look like from the patient perspective (Appendix 1) [18].

In a study carried out by Picker Institute Europe and the University of Oxford where they developed a set of items to measure people experience of integrated care, the “I” statements were used as the framework for development [16]. The study involved a review of evidence, focus groups with patients, a desk review of existing measures, workshops with experts and cognitive testing with patients and healthcare providers. At the end of the process, they suggested a list of 18 statements (“items”) grouped into the six dimensions of integrated care. However, these items have never been validated in a population study.

Based on the dimensions of integrated care model, a consensus was reached among the authors of this paper (CH, MCC and ML) with permission from the first author of the study (JK) [15] to select the most relevant items (n = 13) to be included in the PEICS. In order to appropriately measure all the dimensions of integrated care, we added items (n = 4) from the initial list of “I” statements, for a total of 17 items. The selected items represented all dimensions of the model of integrated care [8]. The PEICS takes approximately 10 minutes to complete. The scale uses three different five-point Likert scales from 0 to 4 adapted from the original items to fit with the PEICS. The total score is the sum of all questions and ranges from 0 to 68, where a higher score indicates better integrated care. Table 1 shows the complete scale.

**Table 1:** PEICS.

Have all your needs been assessed? □ All of my needs have been assessed □ Almost all my needs have been assessed □ Some of my needs have been assessed □ Few of my needs have been assessed □ None of my needs have been assessed Were you as involved as you wanted to be in decisions about your care and support? □ Always □ Often □ Sometimes □ Rarely □ Never Was your family or carer as involved in decisions about your care and support as you wanted them to be? □ Always □ Often □ Sometimes □ Rarely □ Never □ There were no family or carers available to be involved □ I don’t want or I don’t need my family or my carers to be involved Overall, do you feel that your carer/family received support from health and social services as needed? □ Always □ Often □ Sometimes □ Rarely □ Never □ There were no family or carers to support Did health and social care staff tell you what will happen next? □ Always □ Often □ Sometimes □ Rarely □ Never When health or social care staff planned care or treatment for you, did it happen? □ Always □ Often □ Sometimes □ Rarely □ Never Were your care and support reviewed as they should be? □ Always □ Often □ Sometimes □ Rarely □ Never Was your medication thoroughly reviewed as it should be? □ Always □ Often □ Sometimes □ Rarely □ Never Did you know who to contact if you needed to ask questions about your condition or treatment? □ Always □ Often □ Sometimes □ Rarely □ Never If you had questions, could you contact the people treating and caring for you? □ Always □ Often □ Sometimes □ Rarely □ Never Did all the different people treating and caring for you work well together to give you the best possible care and support? □ Always □ Often □ Sometimes □ Rarely □ Never Did health and social care services help you live the life you want? □ Completely □ A lot □ Moderately □ A little □ Hardly Did health and social care staff give you information about other services that are available to someone in your circumstances, including support organisations’? □ Always □ Often □ Sometimes □ Rarely □ Never Was information given to you at the right time? □ Always □ Often □ Sometimes □ Rarely □ Never Was information provided in a way that you could understand? □ Always □ Often □ Sometimes □ Rarely □ Never Could you meet/phone/email a professional when you needed to ask more questions or discuss the options? □ Always □ Often □ Sometimes □ Rarely □ Never If you still needed contact with previous services/professionals, would it be possible? □ Always □ Often □ Sometimes □ Rarely □ Never

### Transcultural adaptation process

The transcultural adaptation process followed the recommendations of Hawkins and Osborne [19]. The process involved four main steps:

1. Forward translation: a native French-speaking professional translator translated the original English-language version into French.
2. Back translation: a native English-speaking professional translator translated the French-language version back into English without having seen the original version.
3. Translation consensus meeting: an expert panel including the two translators, one bilingual patient, four authors of the study (three of them being healthcare providers), and one developer of the measure met to evaluate each translated item. The aim was to clarify and agree upon the translation, to determine if it was comparable to the original English-language version.
4. Cognitive interviewing: Fifteen interviews with patients with chronic conditions seen in primary care were conducted to evaluate the face validity of the French-language version of the PEICS. A one-hour cognitive interview was done with each patient who read each question out loud and then expressed what he or she thought of the question. Over the course of the interviews, the authors of the study discussed and adapted items to improve patient understanding. When the patients reported no difficulty in completing the PEICS, a final version of the translated measure was drafted.

### Validation study

#### Setting and participants

French-speaking patients from the waiting room of two primary care clinics in Quebec, Canada were solicited to complete a questionnaire. The first clinic, including 7 practices, was located in a rural region while the second, including 21 practices, was based in an urban region. Participants had to be: over 18 years of age; a Native French speaker; and affected by at least 1 chronic condition such as diabetes, cardiovascular disease, chronic obstructive pulmonary disease, asthma, hypertension, or other. Pregnant women or patients with an unstable acute condition, an uncontrolled psychiatric or cognitive disease were excluded.

#### Data collection

Two research assistants asked eligible patients to self-complete the questionnaire a first time (T1) while they were in the waiting room of the primary care clinic. The questionnaire included a sociodemographic questionnaire and the French-language version of the Disease Burden Morbidity Assessment (DBMA) [20]; the PEICS; and the Continuity of Care from Multiple Clinicians (CC-MC) [21]. The DBMA (21 items) provides a count of the number of chronic conditions and takes into account patients’ appreciation of the limitations arising from the presence of these chronic conditions. The CC-MC (34 items) measures continuity of care from the patient’s perspective. A moderate correlation was therefore expected (concurrent validity) between the PEICS and three dimensions of the CC-MC: Coordination role (5 items), Comprehensive knowledge of patient (4 items), and Team relational continuity (2 items). Two weeks after the first completion, a research assistant contacted the participants by telephone to complete the questionnaire again (T2), by starting with the first T1 respondents until 50 were completed.

This study was approved by the ethics review boards of the Centre intégré universitaire de santé et de services sociaux du Saguenay–Lac-Saint-Jean and the Centre intégré universitaire de santé et de services sociaux de l’Estrie-CHUS (Quebec, Canada). All participants completed and signed an informed consent form.

#### Analysis

Incomplete questionnaires were excluded. One questionnaire was removed from the analysis due to the lack of coherence between T1 and T2, where a lot of responses passed from ‘never’ to ‘always’ within two weeks. We described the sample using mean and standard deviation (SD) for continuous variables such as age and integrated care, and proportions for categorical variables such as sex, birthplace, first language, marital status, education, occupation, and family income. The data were analysed using SPSS 24.0.

Psychometric properties of the PEICS were calculated by evaluating the internal consistency using Cronbach’s alpha and the test-retest reliability with the intraclass correlation coefficient (ICC). Because the Kolmogorov-Smirnov test demonstrated non-normality of the distribution, the concurrent validity between the PEICS and the three subscales of the CC-MC was calculated with a Spearman’s rank correlation coefficient.

#### Sample size

A minimum sample size of 113 participants was estimated based on detecting a large effect size with α <= .05 and a power of 80% [22]. A total of 159 participants were included in this study, which is higher than expected. For test-retest reliability, a sample size of 50 participants is considered adequate [23].

## Results

**Figure 1:**
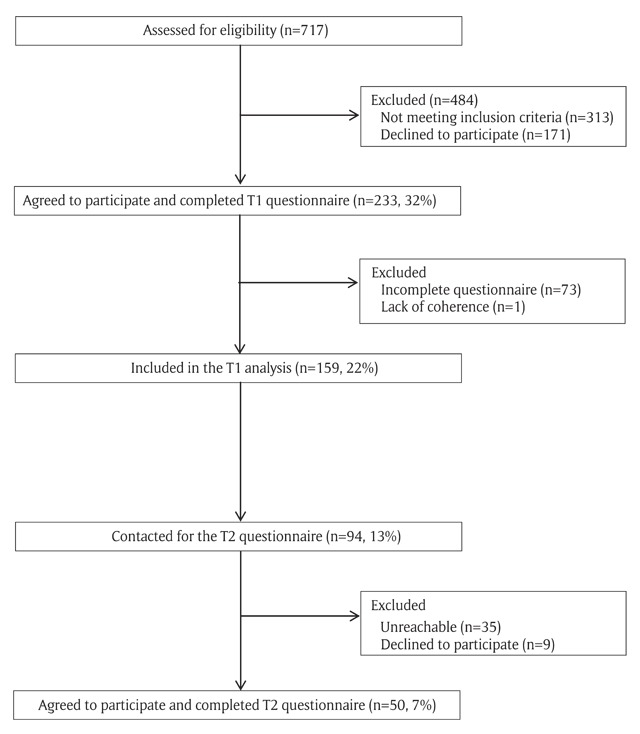
Flow of the patients throughout the study.

A total of 159 participants were included in the study. Figure 1 shows the flow of the patients throughout the study.

Participant characteristics are presented in Table 2.

**Table 2:** Sociodemographic characteristics of the participants (n = 159).

Age: mean (SD)	58 (16)
Number of conditions – DBMA: mean (SD)	4.1 (2.3)
Male: n (%)	53 (33.3)
Education completed: n (%) *5 missing*	
Less than high school	36 (23.4)
Completed high school	45 (29.2)
College	41 (26.6)
University	32 (20.8)
Occupation: n (%) *6 missing*	
Employed	56 (36.6)
Unemployed	24 (15.7)
Retired	73 (47.7)
Annual family income: n (%) *6 missing*	
Less than 20,000 CAD	20 (13.1)
20,000 to 49,999 CAD	59 (38.5)
50,000 CAD or more	74 (48.4)
Marital status: n (%) *1 missing*	
Married/living with a partner	85 (53.8)
Separated, divorced	29 (18.3)
Widowed	21 (13.3)
Single	23 (14.6)

SD: Standard deviation; CAD: Canadian dollar.

Table 3 presents the descriptive statistics of the PEICS as well as its reliability and validity.

**Table 3:** Descriptive statistics and reliability of the PEICS.

Minimum score	Maximum score	Mean (SD)	Internal consistency: Cronbach alpha (95% CI)	Test-retest reliability: ICC (95% CI)

0	68	53 (10)	0.88 (0.85–0.91)	0.81 (0.64–0.90)

SD: Standard deviation; ICC: Intra-class correlation; CI: Confidence interval.

The mean score of the PEICS was 53 out of 68 (SD 10). Cronbach’s alpha for the whole questionnaire was 0.88 (95% CI: 0.85 to 0.91). The intraclass correlation coefficient was 0.81 (95% CI: 0.64 to 0.90). The correlation coefficient between the PEICS and the three subscales of the CC-MC varied from 0.44 to 0.54 (Table 4).

**Table 4:** Concurrent validity between the PEICS and three subscales of the CC-MC.

CC-MC subscale	Concurrent validity: Spearman rank correlation coefficient

Coordination role (5 items)	0.49*
Comprehensive knowledge of patient (4 items)	0.54*
Team relational continuity (2 items)	0.44*

*p ≤ 0.01.

## Discussion

This first validation study of the PEICS showed a high internal consistency and good temporal stability. A moderate correlation was found between the PEICS and three subscales of the CC-MC. These findings were expected because integrated care and continuity of care are related concepts [24], both including dimensions such as communication and cooperation between care providers to ensure that care is connected. The robustness of the measure could be attributed to its development based on a strong model of integrated care [8] and the development of items from a literature review, focus groups with patients, and workshops with experts [8,16].

To our knowledge, the PEICS is the only instrument designed to evaluate all dimensions of the National Collaboration for Integrated Care and Support’s model in the United Kingdom [8]. Few instruments measure integrated care from the patient’s perspective. The Patient Perceptions of Integrated Care (PPIC) developed by Singer *et al.* including 36 items showed good internal consistency (Cronbach’s alpha ranged from 0.62–0.80) and discriminant validity (Cronbach alpha coefficients were generally higher than the interscale correlations) [13]. A 46-item instrument designed by Walker *et al.* [15] also demonstrated excellent internal consistency for 4 subscales out of 5. However, both instruments are too time-consuming to be used in research and clinical practices. On the other hand, the brief IntegRATE instrument included four items and was designed to assess integrated care from the patient perspective, but has never been validated in a large population (>15 patients) [14].

The PEICS could be used to assess the experience of patients with chronic conditions with integrated care among healthcare and social care providers. In clinical settings, such evaluation could provide feedback that leads to improvement. This questionnaire could also be used to measure and evaluate integrated care in various research designs.

### Strengths and limitations of the study

We applied rigorous linguistic and cultural adaptation methods to the questionnaire with the involvement of the first author of the original items. In addition, our study took place in two different regions of Quebec (Canada), increasing the generalizability of the findings. As the first study assessing the validity of the PEICS, no other study is available for comparison. Moreover, our study may not be applicable to patients on the lower end of the socioeconomic status scale, youth, or people without chronic conditions because very few or none of the participants in our study fell into these categories. The 50 respondents for the follow-up questionnaire were not randomly recruited from among all the participants. A response bias could have been introduced by the use of different methods of completion of the questionnaire at T1 (self-administered in the waiting room) and at T2 (by telephone with a researcher assistant). In addition, several questionnaires (n = 73) were excluded because the patients ran out of time to complete the PEICS in the waiting room before their medical visit.

Future studies should consider concurrent validity with other instruments measuring integrated care. In addition, the factorial structure of the scale has to be confirmed in a larger sample. Sensitivity to change over time should also be further considered as well as validity in samples under-represented in this study.

## Conclusion

The PEICS showed good psychometric properties. This scale could be used in a population with chronic conditions followed in primary care to measure patient experience of integrated care, to gather feedback in clinical settings or in a context of research evaluation. Further studies could evaluate its factorial structure, sensitivity to change over time, as well as concurrent validity with other instruments measuring integrated care.

## Additional File

The additional file for this article can be found as follows:

10.5334/ijic.4163.s1 Appendix 1 Statements included in the “I” statements narrative.
