# Supplementary material for: The Patient Experience of Integrated Care Scale: A Validation Study among Patients with Chronic Conditions Seen in Primary Care
Source: Int J Integr Care. 2018 Oct 12;18(4):1. doi: 10.5334/ijic.4163 (PMC6199562; doi:10.5334/ijic.4163)
Supplement: Appendix 1 — Statements included in the “I” statements narrative. [file ijic-18-4-4163-s1.pdf]

Appendix 1. Statements included in the “I” statements narrative.

My goals/outcomes

1. All my needs as a person are assessed.
2. My carer/family have their needs recognised and are given support to care for me.
3. I am supported to understand my choices and to set and achieve my goals.
4. Taken together, my care and support help me live the life I want to the best of my ability.

Communication

5. I tell my story once.
6. I am listened to about what works for me, in my life.
7. I am always kept informed about what the next steps will be.
8. The professionals involved with my care talk to each other. We all work as a team.
9. I always know who is coordinating my care.
10. I have one first point of contact. They understand both me and my condition(s). I can go to them with questions at any time.

Information

11. I have the information, and support to use it, that I need to make decisions and choices about my care and support.
12. I have information, and support to use it, that helps me manage my condition(s).

13. I can see my health and care records at any time. I can decide who to share them with. I can correct any mistakes in the information.
14. Information is given to me at the right times. It is appropriate to my condition and circumstances. It is provided in a way that I can understand.
15. I am told about the other services that are available to someone in my circumstances, including support organisations.
16. I am not left alone to make sense of information. I can meet/phone/email a professional when I need to ask more questions or discuss the options.

Decision making including budgets

17. I am as involved in discussions and decisions about my care, support and treatment as I want to be.
18. My family or carer is also involved in these decisions as much as I want them to be.
19. I have help to make informed choices if I need and want it.
20. I know the amount of money available to me for care and support needs, and I can determine how this is used (whether it’s my own money, direct payment, or a ‘personal budget’ from the council or NHS).
21. I am able to get skilled advice to understand costs and make the best use of my budget.
22. I can get access to the money quickly without over-complicated procedures.

Care planning

23. I work with my team to agree a care and support plan.

24. I know what is in my care and support plan. I know what to do if things change or go wrong.
25. I have as much control of planning my care and support as I want.
26. I can decide the kind of support I need and how to receive it.
27. My care plan is clearly entered on my record.
28. I have regular reviews of my care and treatment, and of my care and support plan.
29. I have regular, comprehensive reviews of my medicines.
30. When something is planned, it happens.
31. I can plan ahead and stay in control in emergencies.
32. I have systems in place to get help at an early stage to avoid a crisis.

Transitions

33. When I use a new service, my care plan is known in advance and respected.
34. When I move between services or settings, there is a plan in place for what happens next.
35. I know in advance where I am going, what I will be provided with, and who will be my main point of professional contact.
36. I am given information about any medicines I take with me – their purpose, how to take them, potential side effects.
37. If I still need contact with previous services/professionals, this is made possible.
38. If I move across geographical boundaries I do not lose my entitlements to care and support.
